# POTS and Antiphospholipid Syndrome: An Unlikely Association

**DOI:** 10.1155/2021/9942668

**Published:** 2021-05-04

**Authors:** Kalvin Zee, Shakaib Qureshi

**Affiliations:** ^1^Rocky Vista University College of Osteopathic Medicine, Shakaib Qureshi, 255 E Center Street, Parker, CO, USA; ^2^Rocky Vista University, Parker, CO, USA

## Abstract

Antiphospholipid syndrome is a rare complication of postural orthostatic tachycardia syndrome. Clinically, the presentation has overlapping symptoms of both diseases, with lightheadedness or syncope when moving from a supine to a standing position as well as blood clots, headache, or pregnancy complications in women. This case presentation involves a 39-year-old patient identified as female who has been diagnosed with POTS and elevated anticardiolipin antibodies.

## 1. Introduction

Postural orthostatic tachycardia syndrome (POTS) is a syndrome of decreased blood flow to the heart when the patient moves from a supine position to a standing position and experiences lightheadedness or syncope as a result. POTS presents with an increase in heart rate during these episodes. Causes of POTS include abnormal blood pressure regulation, impaired nerve function, and autoimmune conditions. Diagnosis is made using a tilt table test that shows a change in blood pressure and heart rate when the patient transitions between lying, sitting, or standing [1].

Antiphospholipid syndrome is an autoimmune disorder characterized by blood clots, miscarriage, or headache due to autoantibodies against phospholipids [2]. Diagnosis requires the presence of lupus anticoagulant, anticardiolipin, or anti-beta-2-glycoprotein I antibodies [2]. The patient is a 39-year-old female with a history of POTS disease and a positive anticardiolipin antibody and antiphospholipid disease symptoms. We hypothesize that there is an association between her POTS disease and antiphospholipid syndrome.

## 2. Case Report

**Figure 1:**
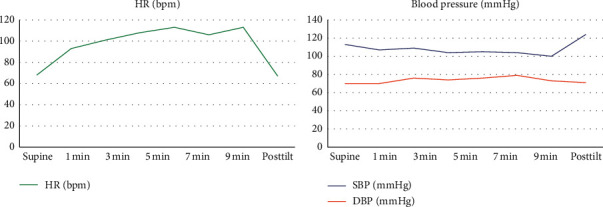
Results of tilt table test.

The patient is a 39-year-old Caucasian female who had recently moved to Utah from Oregon. She presented in clinic with fatigue, postural tachycardia, migraines, and tingling of her extremities. The patient had not been feeling well the previous year when she presented to her primary care physician with a complaint of right-sided supraclavicular adenopathy. The patient underwent imaging and laboratory studies which were all negative. No diagnosis was made at that point. She returned to the clinic twice in the following 2 months with complaints of fatigue, night sweats, tremor, tachycardia while standing, bradycardia while sleeping, dizziness, tinnitus, cold extremities, and tingling throughout her body. During that same year, the patient was also seen in the emergency room multiple times for cervical instability due to degenerative disc disease, and she followed up with a neurosurgeon for chronic cervical disc degeneration. She visited an endocrinologist who ruled out Addison's disease. An MRI of the brain was negative. Ultimately, after undergoing a positive tilt table test with a neurologist, the patient was diagnosed with POTS (Figure 1).

The patient was started on fludrocortisone and improved for 2 weeks before regressing. Repeat basic metabolic panel showed only mild hyperkalemia. However, the patient experienced anxiety at this time, so her medication was switched to midodrine 5 mg BID. The patient stopped taking midodrine after being diagnosed with COVID-19 but resumed the medication after recovery.

The patient has a past medical history of anxiety and has been prescribed citalopram, trazodone, and clonazepam as needed. She also has a history of hypothyroidism, Raynaud's syndrome, and ulnar neuropathy of the right elbow. She had two pregnancies, both of which were complicated by preeclampsia and intrauterine growth restrictions, respectively. Currently, on disability leave, the patient formerly worked as a physician assistant in an orthopedic office. She denies alcohol, tobacco, or other drug use. Family history was noncontributory to her condition.

**Figure 2:**
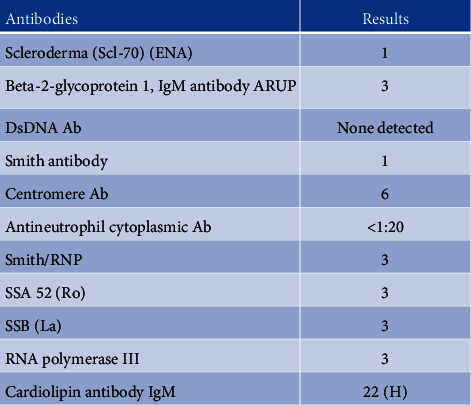
Results of rheumatologic blood tests.

The patient experienced ongoing fatigue, postural tachycardia, headaches, and tingling in her hands and extremities on presentation to this clinic. Her ANA blood test was positive, and she denied any swollen lymph nodes, rash, and respiratory or cardiovascular problems. On exam, her vitals were within normal limits. The remainder of her exam was benign other than a slight extensor weakness of the right forearm attributable to her ulnar neuropathy. There were no positive tender points indicative of fibromyalgia, no joint swelling or effusion, and no apparent synovitis. Recent chemistries drawn showed a qualitative titer of ANA positivity and a positive anticardiolipin antibody IgM of 22 (Figure 2), where the normal range is 0–12. Anti-beta-2-glycoprotein I antibodies were normal with a result of 3. Anticardiolipin antibody IgG and lupus anticoagulant titers were not drawn.

The patient followed up at Stanford Medical Center to confirm her POTS diagnosis and assess the association with antiphospholipid syndrome. She was advised to continue taking aspirin, midodrine, increase fluid intake to 4 L/day, and start the levine exercise protocol to help manage symptoms.

Follow-up titers were drawn 12 weeks later showing an elevated anticardiolipin IgM antibody of 16 (normal range = 0–12). Beta-2 cardiolipin antibodies as well as anticardiolipin IgA and IgG levels were normal. Lupus anticoagulant was negative.

Although the patient does not fulfill the revised Sapporo classification criteria for antiphospholipid syndrome diagnosis, her past obstetric history, past medical history of migraines, and positive anticardiolipin antibodies are suggestive of likely antiphospholipid syndrome. Given her diagnosis of POTS and history of Raynaud's syndrome, we hypothesize that there is an autoimmune correlation between POTS and antiphospholipid syndrome.

## 3. Discussion

A pathogenesis of POTS includes an autoimmune basis with a high prevalence of positive ANA and comorbid autoimmune disorders [3]. Additionally, there has been an association between POTS syndrome and antiphospholipid antibody syndrome [4]. Patients can present with migraine, memory loss, balance disorder, and Raynaud's syndrome. Although POTS is most commonly caused by dysfunctional regulation of blood pressure, there is an association with autoimmune conditions. Among the most common are rheumatoid arthritis and Sjogren's disease [5]. There is also an association between POTS and autoimmune markers gAChR, GPCR, and cardiac lipid raft-associated [3, 6]. It has been hypothesized that an increase in IL-6 inflammatory markers seen in rheumatologic conditions can increase sympathetic drive, thus worsening POTS [4].

Antiphospholipid syndrome has been shown to have numerous cardiac and neurologic manifestations [7]. In some patients, treatment with anticoagulation or antiplatelets improved symptoms [8]. However, it remains controversial whether aspirin is adequate as a prophylactic agent [9], although the patient states that aspirin has been relieving her symptoms. If symptoms persist or return, the use of IVIG to treat antiphospholipid syndrome can be considered [10]. Initially, POTS should be treated nonpharmacologically with increased fluid intake and exercise; however, effective pharmacologic agents include fludrocortisone, DDAVP, and central sympatholytic medications [8].

Limitations to this case include not meeting the threshold for the revised Sapporo classification criteria [11]. The patient does continue to have high anticardiolipin IgM antibodies on two different blood draws 12 weeks apart. However, although the patient has a prior medical history of migraines and a past obstetric history of preeclampsia and intrauterine growth restrictions, neither pregnancy resulted in a premature birth before the 34^th^ week of gestation, and the patient has not had a venous thromboembolic event. This is suggestive of likely antiphospholipid syndrome, but the patient does not meet threshold for that diagnosis at this time.

## 4. Conclusion

POTS has been shown to have an autoimmune basis as a causal mechanism. This case shows a probable relationship between antiphospholipid syndrome and POTS. Although the association between antiphospholipid syndrome and POTS is rare, it is imperative to consider atypical causal mechanisms of diseases.

## Data Availability

No data was used to support this study.
